# Induced hypertension for the treatment of acute MCA occlusion beyond the thrombolysis window: case report

**DOI:** 10.1186/1471-2377-6-46

**Published:** 2006-12-19

**Authors:** Tanya Bogoslovsky, Olli Häppölä, Oili Salonen, Perttu J Lindsberg

**Affiliations:** 1 Department of Neurology, Helsinki University Central Hospital, Helsinki, Finland; 2 Department of Radiology, Helsinki University Central Hospital, Helsinki, Finland; 3 Neuroscience Program, Biomedicum Helsinki, Helsinki, Finland

## Abstract

**Background:**

A minority of stroke patients is eligible for thrombolytic therapy. Small pilot case series have hinted that elevation of incident arterial blood pressure might be associated with a favorable prognosis either in acute or subacute stroke. However, these patients were not considered for thrombolytic therapy and were not followed – up systematically. We used pharmacologically induced hypertension in a stroke patient with middle cerebral artery (MCA) occlusion ineligible for thrombolysis that was followed-up by radiological, clinical and functional outcome assessment.

**Case presentation:**

A patient with acute embolic MCA occlusion producing a large, ischemic penumbra confirmed by perfusion CT was treated by induced hypertension with phenylephrine started within 4 h of admission. Increase in the mean arterial pressure by 20% led to a reduction of neurological deficit by 3 points on the National Institute of Stroke Scale. MRI and CT scans performed during phenylephrine infusion showed the presence of limited subcortical and cortical infarct changes that were clearly less extensive than the perfusion deficit in the brain perfusion CT at baseline, found in the absence of MCA patency. No complications due to induced hypertension therapy occurred. Moderate functional improvement up to modified Rankin scale 2 at follow up took place.

**Conclusion:**

Induced hypertension in acute ischemic stroke seems clinically feasible and may be beneficial in selected normo- or hypotensive stroke patients not eligible for thrombolytic recanalization therapy.

## Background

Blood pressure (BP) increases after the first minutes of acute cerebrovascular occlusion [1] and decreases significantly from admission to 12 hours after thrombolysis especially in case of adequate recanalization [2]. The physiologic reasons for BP elevation, presumably may represent increased sympathetic autonomic nervous activity mediated by cerebral reticular neuronal networks and the medullary vasomotor center that regulate the tone of resistance of vessels of the body. Due to the failure of auto-regulation of cerebral blood flow (CBF) in the ischemic human brain, the CBF is passively dependent on the mean arterial pressure [3]. Thus, the acutely elevated BP may help to maintain a vital level of CBF to support the existing penumbra, and contribute to the preservation of neurological function. Therefore, hypertension may be a risk marker of poor outcome due to initially large infarction or failed vessel recanalization [2] rather than a causative factor of the acute ischemic stroke [4].

Induced hypertension (IH) is a standard treatment for cerebral ischemia in patients with vasospasm after subarachnoidal hemorrhage. Elevation of BP may improve neurological function and CBF in subacute ischemic stroke [5], or alleviate the degree of neurologic dysfunction during acute ischemic stroke [6]. Phenylephrine-induced hypertension (PHE-IH) decreased perfusion deficit by 40% as detected by MRI on the next day [7], suggesting the efficacy of PHE-IH in precluding infarct maturation within the ischemic penumbra.

We report a case where thrombolysis of acute embolic MCA occlusion was not possible due to undocumented onset time of stroke symptoms, but where a large area of cerebral cortex corresponding to ischemic penumbra did not undergo infarction following IH therapy.

## Case presentation

A 72-year right-handed woman with paroxysmal atrial fibrillation (AF), hypertension, coronary artery disease (CAD), asthma and 2 recent transient ischemic attacks was admitted to the emergency department with left hemiparesis and dysarthria that were noticed on awakening 4 h 30 min prior to the admission. She had no anticoagulant therapy. The baseline Barthel index of activities of daily living was 95 and Rankin scale (mRS) was 1. Neurologic examination revealed mild disorientation, left sensomotor hemiparesis, dysarthria, and visual and tactile neglect. The National Institute of Health Stroke Scale (NIHSS) score was 14. The mean BP was 150/72 mmHg, the mean MAP (mean arterial pressure) was 98 mmHg and ECG showed AF.

Immediate brain CT showed a hyperdense right MCA, hypoattenuation of the right lentiform nucleus and insular cortex as well as slight swelling of cortical sulci in the right frontoparietal region. Standard noncontrast CT scanning was performed with a Lightspeed Ultra CT scanner (General Electric Medical Systems; Version 05 MW 14.5.H2_P_M8_G) using the following parameters: 140 kV (posterior fossa/infratentorial)/120 kV (supratentorial), automatic mA (80–150 mA), 512 × 512 image matrix, 23-cm displayed field of view (DFOV), and 5-mm (posterior fossa/infratentorial)/7,5-mm (supratentorial) slice thickness.

**Figure 1:**
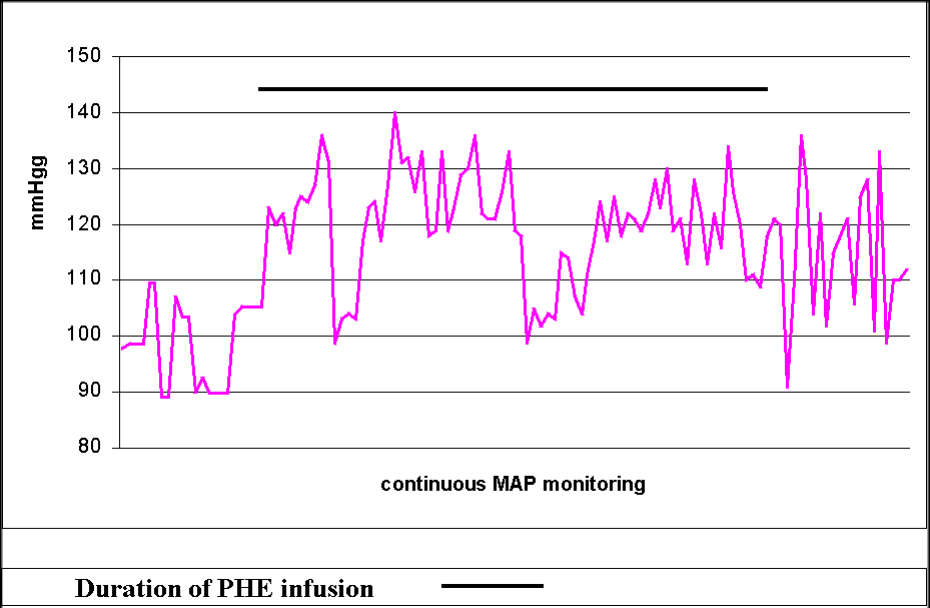
Mean MAP on admission, during induced hypertension (days 1–5) and tapering off (days 6–7).

In CT perfusion examination, the flow- and mean transit time images showed a broad perfusion deficit in the right MCA region. The blood volume chart, however, showed a clearly less significant defect in this region, consistent with the presence of large ischemic penumbra in the cortical cerebral tissue within the MCA territory (Fig 1, top row). CT angiography documented a thrombus of 1 cm in length in the right MCA. Some signs of blood flow were detected distally in the right MCA, presumably resulting from collateral circulation (not shown). For the perfusion CT study, 4 adjacent levels were selected at the level of the basal nuclei. Fifty 5-mm CT sections of continuous (cine) scanning (80 kV, 200 mA) were obtained at every adjacent level, with a total acquisition time of 50 seconds. Computed tomography was initiated 5 seconds after the intravenous rapid infusion (injection rate of 7 ml/s) of 350 mg/ml iodinated nonionic contrast material (Iomeron 350, Bracco). The contrast agent was injected into an antecubital vein with a power injector (Medrad).

A diagnosis of a cardio-embolic right MCA occlusion was thus established. Due to the delay in admission and uncertain timing of symptom onset the patient was not eligible for thrombolysis. In attempt to salvage the obviously large ischemic penumbra, induced hypertension (IH) therapy by phenylephrine (PHE) (Neo-Synephrine 0,1 mg/ml; initial dose 0.5 mg/hour)) and crystalloids 3000 ml/day intravenously was initiated 4 hours after the admission. The goal of 20 % MAP augmentation was achieved within 1 hour after the initiation of the PHE-infusion (Figure 1). During the next 4 days the average MAP was maintained at 120 (range 140-91) mmHg with PHE infusion rate up to 3,5 mg/hour, thereafter PHE was tapered off during 2 days (average MAP 114,5 (range 133-99) mmHg). During PHE infusion the patient had paroxysmal AF with average heart rate 100 beats/min. The antihypertensive medication (Athenolol and Lozartan) that the patient had before the admission was stopped at the time of initiation of IH.

**Figure 2:**
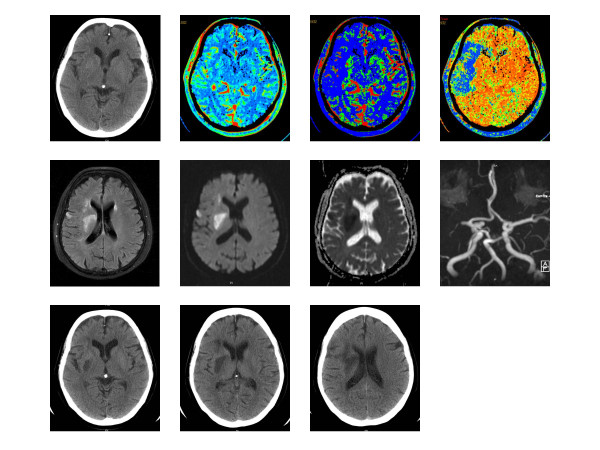
**Top row, left**: Admission CT showing hypoattenuation of the right lentiform nucleus and insular cortex as well as slight swelling of cortical sulci in the right frontoparietal region. **Top row, the third and the fourth pictures**: The perfusion CT on admission, with flow and mean transit time CT showing a broad perfusion deficit in the right MCA region. **Top row, the second picture**: The volume chart shows only a small deficit in the right MCA region, consistent with the presence of a large ischemic penumbra in the cortical cerebral tissue. **Middle row**: The follow-up brain MRI showing the presence of a limited subcortical infarct on the distal region of the right lentiform nucleus and corona radiata. Signs of only a subtle cortical infarction are seen. **From left to right**: FLAIR MRI, T2-diffusion-weighted MRI, apparent diffusion coefficient. **Middle row, right**: The persistence of the right MCA occlusion on the follow up MRA. **Bottom row**: Day 2 CT showed that the extent of the subcortical infarct was substantially smaller than that of the perfusion deficit found in the CT perfusion brain scan obtained on admission.

Four hours after the initiation of MAP augmentation the gaze deviation and the left leg weakness were improved and NIHSS was 12. Sixteen hours after the MAP augmentation the left facial weakness and hemiparesis were improved, although the patient was still slightly somnolent, and had moderate dysarthria and neglect. The NIHSS improved to 11. The brain CT and MRI scans performed 21 h and 26 h after admission showed the presence of a limited subcortical infarct on the distal region of the right lentiform nucleus and corona radiata. There were only subtle signs of a cortical infarction. Moreover, the extent of the subcortical infarct was substantially smaller than that of the perfusion deficit found in the brain perfusion CT on admission (Fig. 2, bottom row). MRA showed the persistence of the right MCA occlusion (Fig. 2, middle row).

During the following days the patient experienced a mild improvement on her level of alertness and in the left hemiparesis, with less significant improvement in the severity of dysarthria and neglect. Antithrombotic treatment with sodium daltreparin followed by warfarin was instituted. When discharged to the rehabilitation hospital, the patient had a NIHSS of 7. At the 3-month control visit she was independent in main activities of daily living, but needed a walking device when strolling outside. The uncontrasted control brain CT showed the previous right MCA infarct without any new signs of ischemia (not shown). NIHSS was 4; Barthel index was 80 and mRS 3. At 10-month interview she lived at home and walked independently, Barthel index was 90 and mRS 2.

## Conclusion

Many stroke patients are not eligible for thrombolysis due to delay in the admission or unclear timing of symptom onset. Still, there may be a therapeutic window for salvaging of brain tissue through improvement of CBF in the ischemic penumbra, which seems to exist well beyond the sofar approved 3-hour window.

This case study suggests the potential utility of IH in the treatment of acute stroke beyond the conventional thrombolytic treatment window. Studies have documented the existence of an ischemic penumbra up to 24 hours after stroke onset the and therapeutic time window for salvaging brain tissue may be substantially longer [8].

Sparing of the ischemic penumbral brain tissue in the present case was confirmed by the follow-up MRI and supported by the relatively good functional outcome. The spontaneous clot lysis or sudden improvement of collaterals to the occluded MCA territory seems unlikely because the original vascular occlusion as well as collateral flow remained essentially identical on the follow-up MRA. A degree of functional improvement was achieved already acutely upon increasing the average MAP by 20%.

Although we cannot confirm that it was PHE-IH that was crucially responsible for the diversion toward a benign outcome, our past experience of nonrecanalized acute MCA occlusions in elderly patients attests of a much more extensive infarct maturation in the MCA territory, especially in cases where BP drops during the first night following the admission. In accordance with previous reports of using IH [9] this study demonstrates relative safety of PHE- IH, the patient was closely monitored, and no evidence of cardiac ischemia were seen, the chest x ray did not reveal any signs of pulmonary edema, and no hemorrhagic transformation on CT scans was observed.

In conclusion, PHE-IH seems to be feasible and could be beneficial in selected stroke patients within 6 hours from stroke symptom onset. A prospective, randomized study of PHE-IH could be warranted to examine whether PHE-IH can salvage ischemic brain tissue in acute, documented cerebrovascular occlusions that are not amenable for thrombolytic therapy even though ischemic penumbra still exists.

## Abbreviations

MCA-middle cerebral artery

MAP-mean arterial pressure

BP-blood pressure

IH-induced hypertension

PHE-phenylephrine

NIHSS-National Institute of Health Stroke Scale

PHE-IH phenylephrine-induced hypertension

CBF-cerebral blood flow

CAD-coronary artery disease

AF-atrial fibrillation

mRS-modified Rankin scale

## Competing interests

The author(s) declare that they have no competing interests.

## Authors' contributions

TB drafted the manuscript and made a contribution to design, analysis and acquisition and interpretation of data. PL made a contribution to design and conception of the study and participated in the analysis of data, and revised the manuscript that led to the final approval of the current submission. OH and OS were involved in the acquisition of clinical data and in the interpretation of data.

## Pre-publication history

The pre-publication history for this paper can be accessed here:
